# Medical Fashions in the Nineteenth Century; Including a Sketch of Bacterio-Mania and the Battle of the Bacilli

**Published:** 1884-03

**Authors:** 


*Medical Fashions in the nineteenth century; including a sketch of Bacterio-mania and the battle of the Bacilli.* By E. T. Tibbits, M.D. Lond. London: H. K. Lewis, 1884.

Following Dr. Hare's example, Dr. Tibbits has written a booklet upon the mutability of medical fashion—fashions both of theory and of practice. In the course of some seventy pages he firstly touches briefly upon a variety of topics, such as bleeding, mercury, the employment of remedies in excessive and in minute doses, alcohol, anæsthetics and so forth, while the latter half of the book is occupied with his views upon the germ theory of disease, or what he is pleased to call bacterio-mania, and the battle of the bacilli.

It is not so stated, but we presume that the text is that of an address to a local society afterwards amplified sufficiently to make a small book. It would be uncharitable to suppose that a physician of Dr. Tibbits' position would, under any other circumstances, put into circulation a work so flimsy in substance and so slip-shod in composition. Instead, therefore, of submitting the book to a formal review, we will let the author express his views upon one or two topics in his own words, as being the fairest method of showing the quality of the information he affords us.

Speaking of anæsthetics, he says:—" Reference may be made to the comparatively recent use of anæsthetics during childbirth. . . . to produce a kind of intoxication and sometimes complete insensibility. Whether I am right or not I cannot help thinking that the invariable use of an anæsthetic in this manner is a most pernicious custom for many reasons."

Speaking of the physiological action of digitalis, he says:—" I believe that the so-called arterial tension supposed to be brought about by the action of digitalis has frequently been simple distension of the blood-vessel, *i.e.*, relaxation (?) of its muscular coat, plus diminished frequency of cardiac pulsations. The 'increased arterial tension,' said to be produced by digitalis, was regarded as a phenomenon of a tonic nature, and finally this drug was classed with stimulants, such as alcohol, ether, ammonia and the like, and in this category it is now placed."

His views on the etiology of typhoid fever are these:—" Insufficient or improper food, exposure to cold, great bodily or mental depression or exhaustion; one or all may in some instances constitute the actual *exciting* cause of a serious attack of typhoid fever."

Lastly, his opinions upon the germ theory of disease may be deduced from this brief sentence:—" That the air contains much organic matter and many individual organisms is beyond the region of dispute, but that such visible organisms have anything whatever to do with disease is pure hypothesis."

Dr. Tibbits' experience with the "wool-sorters' disease," which occurs prominently in Bradford, has led him to take a special interest in bacilli, as the causes or concomitants of diseases like anthrax and tubercle, and nearly half of this book is occupied with a discussion upon what he terms the battle of the bacilli. Although none would be convinced and few in any degree influenced by his arguments, still this part of the book is written with a more intimate acquaintance with the subject than is relatively displayed in the other sections, and it is at least worth reading.

Dr. Tibbits promises soon another somewhat similar work, called "Mores Medici;" we hope it will help to sustain his reputation by being a contribution to medical literature more solid and more scientific than "Medical Fashions."

